# Randomized trials published in some Chinese journals: how many are randomized?

**DOI:** 10.1186/1745-6215-10-46

**Published:** 2009-07-02

**Authors:** Taixiang Wu, Youping Li, Zhaoxiang Bian, Guanjian Liu, David Moher

**Affiliations:** 1 Chinese Cochrane Centre, Chinese Evidence-Based Medicine Centre, International Clinical Epidemiology Network (INCLEN), Local Resource and Training Center, West China Hospital, Sichuan University, Chengdu, PR China; 2 School of Chinese Medicine, Hong Kong Baptist University, Hong Kong, PR China; 3 Ottawa Methods Centre, Ottawa Health Research Institute, The Ottawa Hospital, General Campus, Critical Care Wing (Eye Institute), Smyth Road, Ottawa, Ontario K1H 8L6, Canada

## Abstract

**Background:**

The approximately 1100 medical journals now active in China are publishing a rapidly increasing number of research reports, including many studies identified by their authors as randomized controlled trials. It has been noticed that these reports mostly present positive results, and their quality and authenticity have consequently been called into question. We investigated the adequacy of randomization of clinical trials published in recent years in China to determine how many of them met acceptable standards for allocating participants to treatment groups.

**Methods:**

The China National Knowledge Infrastructure electronic database was searched for reports of randomized controlled trials on 20 common diseases published from January 1994 to June 2005. From this sample, a subset of trials that appeared to have used randomization methods was selected. Twenty-one investigators trained in the relevant knowledge, communication skills and quality control issues interviewed the original authors of these trials about the participant randomization methods and related quality-control features of their trials.

**Results:**

From an initial sample of 37,313 articles identified in the China National Knowledge Infrastructure database, we found 3137 apparent randomized controlled trials. Of these, 1452 were studies of conventional medicine (published in 411 journals) and 1685 were studies of traditional Chinese medicine (published in 352 journals). Interviews with the authors of 2235 of these reports revealed that only 207 studies adhered to accepted methodology for randomization and could on those grounds be deemed authentic randomized controlled trials (6.8%, 95% confidence interval 5.9–7.7). There was no statistically significant difference in the rate of authenticity between randomized controlled trials of traditional interventions and those of conventional interventions. Randomized controlled trials conducted at hospitals affiliated to medical universities were more likely to be authentic than trials conducted at level 3 and level 2 hospitals (relative risk 1.58, 95% confidence interval 1.18–2.13, and relative risk 14.42, 95% confidence interval 9.40–22.10, respectively). The likelihood of authenticity was higher in level 3 hospitals than in level 2 hospitals (relative risk 9.32, 95% confidence interval 5.83–14.89). All randomized controlled trials of pre-market drug clinical trial were authentic by our criteria. Of the trials conducted at university-affiliated hospitals, 56.3% were authentic (95% confidence interval 32.0–81.0).

**Conclusion:**

Most reports of randomized controlled trials published in some Chinese journals lacked an adequate description of randomization. Similarly, most so called 'randomized controlled trials' were not real randomized controlled trials owing toa lack of adequate understanding on the part of the authors of rigorous clinical trial design. All randomized controlled trials of pre-market drug clinical trial included in this research were authentic. Randomized controlled trials conducted by authors in high level hospitals, especially in hospitals affiliated to medical universities had a higher rate of authenticity. That so many non-randomized controlled trials were published as randomized controlled trials reflected the fact that peer review needs to be improved and a good practice guide for peer review including how to identify the authenticity of the study urgently needs to be developed.

## Background

A systematic review has found that some countries, including China, report unusually high proportions of positive results in published randomized controlled trials (RCTs) [1]. One factor that may contribute to this effect is selection bias. It has been shown that inadequate randomization approaches result in estimates of treatment effects that are more favorable than those derived from properly randomized trials [2,3]. A systematic review conducted by Gu *et al*. [4] provides insight into this methodological shortcoming in trials conducted in China. In an effort to evaluate the benefits and harms of Chinese medicinal herbs in the treatment of measles, they identified a total of 28 reports of randomized trials that appeared to meet their eligibility criteria. As part of their review they conducted telephone interviews of 19 corresponding or first authors and discovered that the study authors had used inappropriate methods to generate the random sequence list and had not adequately concealed the participant allocation. These studies could therefore not be regarded as authentic randomized trials. Although this is a disturbing result, it is difficult to assess whether it represents a misunderstanding on the part of a few researchers about fundamental methodological issues in the conduct of randomized trials, or whether it reflects a larger problem prevalent among trials conducted throughout China. In this study, we therefore aimed to investigate the adequacy of randomization of RCTs published in Chinese journals and the extent of the authenticity of randomized trials using a large cross-sectional sample of the Chinese literature.

## Methods

### Selection of studies

Twenty-one investigators searched the China National Knowledge Infrastructure electronic database for the period January 1994 to June 2005, for trials written by Chinese researchers and published in Chinese journals on a convenient sample of 20 common diseases. Any trial described by the authors as an RCT or that claimed to have used random sequence generation or allocation concealment was considered eligible.

To identify studies from the initial selection for further examination, we scanned the titles, abstracts, and keywords of every record retrieved for the phrases 'randomized controlled trial', 'randomly allocated', and 'random method was used'. The search strategy is given in Appendix 1.

### Telephone interviews

Telephone interviews were conducted by trained collaborators with the first author of each article or, if he or she was unavailable, a co-author. The interview questions were designed to determine the quality of the author's understanding of the principles of randomization. If an author had used an inadequate method of randomization but deemed it to be correct, he or she was judged not to understand the principles of randomization. If the author claimed that he or she had known that the method of randomization used was incorrect, or that he or she had been unable to strictly control the allocation according to a generated random sequence, the author was judged to have intentionally misidentified the study as an RCT.

### Quality control

Before conducting the telephone interviews, investigators were given training in communication and interviewing skills and in the design, conduct, and critical appraisal of RCTs, especially with regard to approaches to the randomization of trial participants. The interview results were recorded in a specially designed form intended to capture publication information, randomization approach, whether the author knew or did not understand correct methods for random sequence generation, concealment of the subsequent sequence, and funding source.

### Criteria for randomization

The survey questionnaire is provided in Appendix 2. Randomization criteria refer to Cochrane reviewer's handbook 4.2.5, Appendix 5a [5]. A randomization sequence generated from a random number table, calculator or computerized random-number generator was considered authentic. Coin-tossing or drawing straws in the presence of the participant to decide which group he or she would be assigned to were considered ineligible randomization techniques. Allocation of participants according to date of birth, or their hospital record number, or the date on which they were invited to participate (for example, an odd or even day) was not considered adequate; studies that used these allocation methods were excluded from our sample.

### Data analysis

The percentage of 'authentic' RCTs as opposed to claimed (self-described) RCTs was calculated and stratified by: (1) location where the trial was conducted; (2) type of intervention (traditional Chinese medicine versus conventional medicine); (3) purpose of the trial with respect to pre-market drug trial; (4) level of institution; we compared hospitals affiliated to medical universities versus other level 3 hospitals; and (5) funding sources (that is, government or other official organization). With respect to level of institution, in China, hospitals are classified into three grades according to their level and size, the highest level being level 3; generally, hospitals affiliated to medical universities, and provincial hospitals, are level 3; others are classified as level 2 or below. Outcomes included: authentic RCT; multiple versions of a published paper; authors who could not be contacted; authors who refused to answer questions; authors who incorrectly claimed non-RCTs as RCTs due to lack of knowledge of RCT methodology; and, authors who intentionally claimed non-RCTs as RCTs. We used a histogram to present a trend of the number of authentic RCTs published over time. We used relative risk (RR) with 95% confidence interval (CI) to estimate the differences of numbers of apparent and real RCTs between different hospitals, traditional Chinese medicine (TCM), and conventional medicine (CM).

## Results

**Figure 1:**
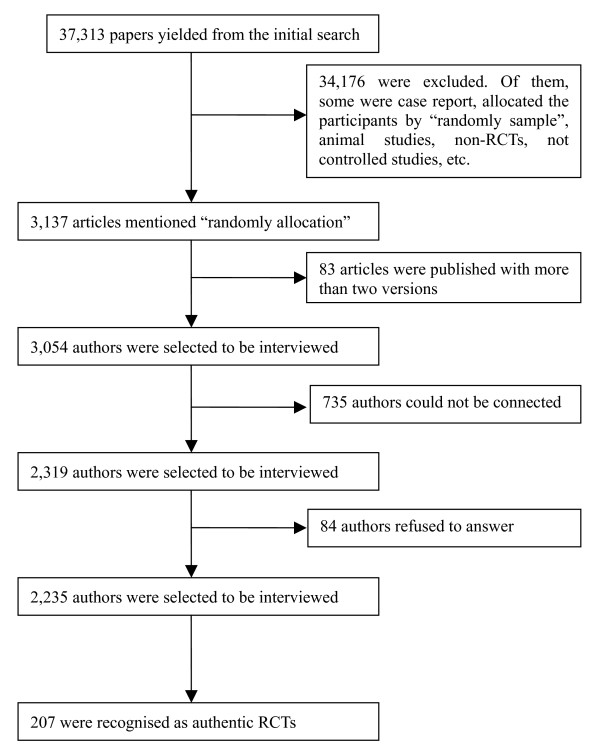
**The flow chart used for including and identifying the trials**.

The search strategy yielded 37,313 records (Figure 1). After screening for keywords we identified 3137 self-described RCTs. Of these, 83 (2.6%) were excluded on the grounds that they had been published in more than two versions in different journals, leaving 3054 articles selected for author interview. All of the included articles and journals were published in Chinese and peer reviewed. The authors of 735 (24.1%) of these could not be contacted, and a further 84 (2.8%) authors refused to answer our questions. Of the remaining 2235 reports, only 207 were found to be authentic RCTs on the basis of their authors' responses to the interview questions (6.8%, 95% CI 5.9–7.7, in 207 out of 2235 as those studies which the authors cannot be contacted were considered critically as unauthentic RCTs, and 9.3%, 95%CI 8.3–10.3 in 207 out of 3054, respectively). Of these, 103 (7.3%) had examined TCM interventions and 104 (6.4%) had examined CM interventions.

### Stratification by type of research setting

An analysis stratified according to type of research setting yielded striking results. Researchers at medical universities or college-affiliated hospitals authored 713 of the self-described RCTs (22.7%). Of these, 30 (4.2%) were found to be the second or third version of a report published in another journal or journals; 162 (23.7%) authors of the original studies could not be contacted, and a further 18 (2.6%) authors refused to participate. The remaining 128 studies were identified as authentic RCTs on the basis of the author interview (18.7%). All of the articles reported results about pre-market drug randomized clinical trials and 51.6% of trials supported by government and other official organizations were identified as authentic.

Authors at level 3 hospitals or medical institutes authored 495 (15.8%) of the self-described RCTs in our sample. Twenty-seven (5.5%) of these were found to be the second or third versions of previously published reports. The original authors of 103 (22.0%) of these reports could not be contacted, and 13 (2.8%) were uncooperative. A total of 55 studies conducted at level 3 hospitals were deemed on the basis of the author interview to be authentic RCTs (11.8%). All of the randomized clinical trials of pre-market drugs were authentic, and of the trials supported by government and other official sources, 56.3% were authentic.

Researchers at level 2 and lower-level hospitals authored 1929 (61.5%) of the self-described RCTs in our sample. Of these, 26 studies (1.3%) had more than two versions. The authors of 470 (24.7%) of the studies from the level 2 group could not be contacted for interview, and 53 (2.8%) refused to cooperate. Only 24 studies conducted at class 2 or lower-level hospitals were deemed authentic RCTs (1.3%); of these, only one was a trial of a pre-market drug, and one had received funding from an official source.

Thus, self-described RCTs conducted at university or college-affiliated hospitals were significantly more likely to be authentic than those conducted at level 3 hospitals and medical institutes (RR 1.58, 95% CI 1.18–2.13) and those conducted at level 2 and lower level hospitals (RR 14.42, 95% CI 9.40–22.10). Similarly, studies conducted at level 3 hospitals were more likely to be authentic than those conducted at level 2 and lower-level hospitals (RR 10.18, 95% CI 6.23–16.63).

### Stratification by category of intervention

Of the 713 RCTs conducted at medical-university or college-affiliated hospitals, 331 (22.8%) examined TCM interventions while 382 (22.7%) examined CM interventions. Of these, 11 (3.3%) TCM reports and 19 (5.0%) CM ones were the second or third version of previously published reports. The authors of 75 (23.4%) of the TCM reports and 87 (24.0%) of the CM reports could not be reached, and the authors of 12 (3.8%) of the TCM papers and 6 (1.7%) of the CM papers were uncooperative. Sixty-nine TCM studies (21.6%) and 59 CM studies (16.3%) were considered authentic RCTs; there was no statistically significant difference in rates of authenticity between trials with respect to type of intervention (RR 1.27, 95% CI 0.93–1.74). All of the RCTs of pre-market drugs were authentic, regardless of whether they were classified as TCM or CM. Similarly, there was no statistically significant difference in rates of authenticity between the TCM and CM categories with respect to funding source (RR 1.33, 95% CI 0.97–1.81; *P *= 0.08 (other source-supported projects), and RR 1.24, 95%CI 0.67 to around 2.30; *P *= 0.49 (government-supported projects, respectively)). Among the 62 projects funded by government and other official sources, 54.3% of the TCM trials were authentic and 43.8% of the CM were authentic; there was no statistically significant difference in authenticity between TCM and CM fields (RR 1.27, 95% CI 0.93–1.74).

Of the 495 self-described RCTs conducted at level 3 hospitals, 192 (13.2%) involved TCM interventions and 303 (19.1%) CM interventions; only 55 of these 495 studies proved to be authentic RCTs, of which 23 (12.4%) and 32 (11.3%) concerned TCM and CM interventions, respectively. Of these reports, 7 (3.6%) TCM studies and 20 (6.6%) CM studies were the second or third version of previously published reports. The authors of 28 (15.1%) TCM studies and 75 (26.5%) CM studies could not be contacted by telephone, and the authors of 6 (3.2%) TCM studies and 7 (2.5%) CM studies refused to answer our questions.

There was no statistically significant difference in the likelihood of authenticity in the level 3 institutions associated with the category of interventions (RR 1.10, 95% CI 0.67–1.82). Five trials in each of the TCM and CM categories were of pre-market drugs; all were deemed authentic RCTs. The rate of authenticity among government and other officially supported projects was 100% in the TCM category and 30% in the CM category.

### Rates of authenticity for different levels of hospital according to category of intervention

Studies conducted at university or college-affiliated hospitals had higher rates of authenticity than studies conducted at class 3 and level 2 hospitals. This difference was statistically significant for both TCM and CM studies (RR 1.68, 95% CI 1.09–2.61, and RR 17.47, 95% CI 9.35–32.64 in the TCM field, and RR 1.46, 95% CI 0.98–2.17, RR 12.53, 95% CI 6.96–22.54 in the CM field, respectively).

Studies conducted at level 3 hospitals and institutes had higher rates of authenticity in comparison with class 2 or lower hospitals. This difference was statistically significant for both TCM and CM studies (RR 11.67, 95% CI 5.58–24.40 for TCM studies, and RR 8.59, 95% CI 4.57–16.15 for CM studies).

### Authors' methodological understanding

We found that 115 authors (5.1%) had a good understanding of randomization methods but still claimed that their non-RCTs were RCTs. Of these, 88 (8.2%) had reported on TCM interventions and 27 (2.3%) on CM interventions. We found that 1913 authors (85.6%) did not fully understand the principles of randomization when they incorrectly claimed that their trials were RCTs. Of these 1913 authors, 882 (82.2%) reported on TCM interventions and 1031 (88.7%) on CM interventions. Of course, we cannot judge whether the authors who could not be contacted or refused to answer our questions had a good understanding of randomization methodology.

**Figure 2:**
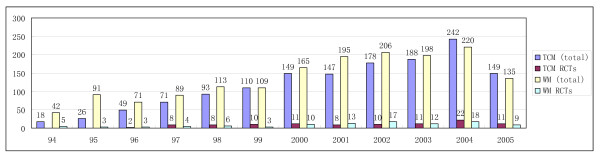
**Total numbers of authors claiming randomized control trials and authentic randomized control trials in traditional Chinese medicine and conventional medicine fields for each year**.

The results of our stratified analysis are given in Additional file 1. A further result, depicted in Figure 2, was that rates of authenticity (as defined by the use of adequate allocation methodology) among self-described RCTs of both TCM and CM studies have been increasing over the past 10 years.

## Discussion

Unethical incomplete reporting of research methods and process providing a misleading explanation of randomization by referring to a non-randomized study as an RCT is a worldwide problem [6-12].

One of the first quality assessments of RCTs published in Chinese journals was conducted in 1999 by Xu [13] and Tang [14] and similar articles have followed more recently [15-32]. These reports identified inadequate or poorly described randomization and a failure to conceal patient allocation as among the critical problems with RCTs conducted and published in China. However, all of these evaluations were based on the methodological claims made in the articles themselves, and some investigators concluded that the number of authentic RCTs has begun to rise [20-29]. Only one research group [32] contacted the original authors of self-described RCTs to investigate their methodological approach; they found that in a sample of 77 self-described RCTs, only two articles were authentic RCTs and two were 'quasi-RCTs'.

Our study used a broad sample of published studies to determine that only about 7% of self-described RCTs published in Chinese medical journals met methodological criteria for authentic randomization. This extremely high proportion of 'false' RCTs contaminates medical information and represents a waste of resources. Data from falsely reported RCTs have the potential to mislead healthcare providers, consumers and policy-makers. Systematic reviewers also need to be alert to the risk of bias in RCTs published in some Chinese journals. It is insufficient to include these reports in a systematic review solely on the basis of their authors' claims; a careful examination of the methods of each RCT must be undertaken in the selection process for a literature review.

Our finding that only half of the projects supported by government or other official organizations were authentic RCTs suggests that improved and strengthened supervision and monitoring of funded projects is needed. We also found an association between authenticity and level of institution, the highest rate of authenticity occurring among studies conducted at university-affiliated hospitals, and the lowest rate occurring among level 2 or lower hospitals. Unfortunately, most of the self-described RCTs (62%) were published by level 2 or lower hospitals.

Our finding that most authors (85.6%) did not have a clear understanding of the methodological principles of randomized trials but claimed their non-RCTs as RCTs, and that some authors (5.1%) had claimed non-RCTs to be RCTs despite their understanding of these principles reflects two problems: first, a lack of knowledge of proper trial design and, second, a disregard for the scientific and social responsibility to use proper trial design. There is an urgent need to educate Chinese clinicians and researchers in clinical trial methodology and reporting [26].

We also found that trials for pre-market drugs included in our study were conducted according to sound methodological principles. This is explained by the fact that the performance of any trial for a pre-market drug must be registered and approved by the Chinese State Food and Drug Administration (SFDA), which releases licenses only for those clinical trials that are designed according to the good clinical practice guidelines.

We supposed that most real RCTs or high quality studies were published in international journals, and most pre-market drug clinical trials had not been published due to the fact that the priority purpose of conducting a clinical trial for drug makers is to get approval from SFDA rather than publishing the results. It is estimated that 1250 applications for authorization of new trials and new drug applications are received each month [33], so, most trials of pre-market drugs were not published in Chinese journals. Some key Chinese journals were not included in our investigation, which may have caused this skewness. However, we have not conducted further investigations to obtain evidence to support this hypothesis.

The limitations of our study included our inability to contact a number of authors (nearly 25%) and the refusal of another 3% to answer our questions. However, given that the rate of authentic RCTs among self-described RCTs was very low, we do not believe that these missing data would significantly affect our findings. Further analysis is warranted to examine the effects of faulty methodology in clinical trials.

Only principal investigators were interviewed, which was another limitation as it is possible that in some cases they did not have a detailed knowledge of the randomization processes used in their trial. This is likely to be a minority of cases. The interviews were conducted by medical students who although trained may have sometimes expressed themselves poorly due to inexperience.

## Conclusion

Most of the articles that mentioned 'randomly allocated patients' of the research were not real RCTs. Most authors of these articles misunderstood the concept of randomization but allocated patients optionally by authors, some authors claimed their non-RCTs to be RCTs despite them understanding the principles of trial design. Of the included studies, randomized controlled clinical trials of pre-market drugs were well designed, and trials conducted in university-affiliated hospitals and high level hospitals had a higher rate of authentic RCTs. Few articles where the authors came from low level hospitals may be considered authentic RCTs.

Education regarding both methodology and social responsibility for healthcare providers who are potential authors of research articles is urgently needed in China.

## Abbreviations

CI: confidence interval; CM: conventional medicine; RCT: randomized control trial; RR: relative risk; SFDA: State Food and Drug Administration; TCM: traditional Chinese medicine

## Competing interests

The authors declare that they have no competing interests.

## Authors' contributions

TW designed and organized the study, trained the investigators, developed the study protocol and drafted the article. YL organized the investigators and, together with TW and ZB contributed to the development of the article. GL conducted the statistical analysis. YL was responsible for the grant application for this research. DM contributed to the organization of the whole project and was responsible for the development of the article.

## Appendix 1: Search strategy

#1 common cold

#2 upper respiratory tract infections

#3 sore throat

#4 bronchitis

#5 measles

#6 pneumonia

#7 myocarditis

#8 hypertension

#9 unstable angina pectoris

#10 angina pectoris

#11 heart failure

#12 peptic ulcer

#13 iron deficiency anemia

#14 esophageal cancer

#15 lung cancer

#16 nephrotic syndrome

#17 prostatic hyperplasia

#18 psoriasis

#19 ovary cancer

#20 icterohepatitis

#21 #1~#21/or

#22 RCT

#23 RCT

#24 randomly allocated

#25 #22~#24/or

#26 #21 AND #25

## Appendix 2: Outline for telephone interviews

1. Introduce yourself and state your purpose: How do you do? I am a student of West China Medical Center, Sichuan University and am doing a review about ['intervention name'] in the treatment of ['condition name']. The purpose of my study is to compare the effects of various randomization methods. I have searched out a paper that you published in [time, journal]. Could you please tell me about the method that was used in this trial?

2. If the subject cannot describe the method clearly, change the question like this: Could you please tell me, when a new participant enrolled, how did you decide which group the participant should be allocated to?

3. If there is any problem with the first author, the second author or others should be interviewed.

4. The next two questions aim to understand the category of funding support for the investigated study. You can select one of them or both. (1) Was your study funded by government or any other source? (2) Did your study concern new drug development?

5. A judgment should be made immediately as to whether or not the trial is an authentic RCT. If it is judged as authentic, the next questions aim to understand the status of allocation concealment. (1) Do you know allocation concealment? If so, please clarify. (2) Did you use any method to mask the allocation sequence? If any, please clarify.

6. The question aims to understand the validity of blinding. Pay particular attention to whether the result assessor was blinded or not. Please tell me who was blinded in your trial?

7. Do not forget to say 'thank you'.

8. Finally, you need to judge whether the subject understands the proper methods of randomized trials. If someone insists that the method of randomization was correct when actually it was wrong or ineligible, he or she should be judged as not understanding. If someone claims 'I know we did not perform the trial well enough' or 'It was impossible to perform a completely correct RCT', and so on, he or she should be judged as having a good understanding but knowingly claiming a non-RCT to be an RCT.

9. Record all of your findings on the form.

## Supplementary Material

Additional file 1 **Table S1**. Characteristics of self-described randomized control trials, stratified by level of institution, funding source, and category of intervention. Click here for file
