# Supplementary material for: Randomized trials published in some Chinese journals: how many are randomized?
Source: Trials. 2009 Jul 2;10:46. doi: 10.1186/1745-6215-10-46 (PMC2716312; doi:10.1186/1745-6215-10-46)
Supplement: Additional file 1 — Table S1. Characteristics of self-described randomized control trials, stratified by level of institution, funding source, and category of intervention. [file 1745-6215-10-46-S1.doc]

| Table S1: Characteristics of self-described RCTs, stratified by level of institution, funding source, and category of intervention | | | | | | | | | | |
| --- | --- | --- | --- | --- | --- | --- | --- | --- | --- | --- |
|  | | **Total, n (%)** | | | **TCM, n (%)** | | | **CM, n (%)** | | |
| Medical university or college-affiliated hospitals | | | | | | | | | | |
| Self-described RCT | | 713 | | (22.7%, 713/3137) | 331 | (22.8%, 331/1452) | | 382 | (22.7%, 382/1685) | |
| Multiple versions of publication | | 30 | | (4.2%, 30/713) | 11 | (3.3%, 11/331) | | 19 | (5.0%, 19/382) | |
| Author could not be contacted | | 162 | | (23.7%, 162/(713−30)) | 75 | (23.4%, 75/(331−11)) | | 87 | (24.0%, 87/(382−19)) | |
| Author refused to answer | | 18 | | (2.6%, 18/(713−30)) | 12 | (3.8%, 12/(331−11)) | | 6 | (1.7%, 6/(382−19)) | |
| Authentic RCTs | | 128 | | (18.7%, 95% CI 15.7~21.5; 128/(713−30)) | 69 | (21.6%, 95% CI 16.5~25.4; 69/(331−11)) | | 59 | (16.3%, 95% CI 12.5~20.3; 59/(382−19)) | |
| Likelihood of authenticity, TCM vs CM | |  | |  | RR 1.33, 95% CI 0.97–1.81; P=0.08; (69/(331−11))/(59/(382−19)) | | | | | |
| Study concerned pre-market drug | | 37 | |  | 22 |  | | 15 |  | |
| Study concerned pre-market drug and was authentic RCT | | 37 | | (100%, 37/37) | 22 | (100%, 22/22) | | 15 | (100%, 15/15) | |
| Study was funded by government or other official source | | 62 | |  | 46 |  | | 16 |  | |
| Study was funded by government or other official source and was authentic RCT | | 32 | | (51.6%, 95% CI 39.2~64.1; 32/62) | 25 | (54.3%, 95% CI 40.0~68.7; 25/46) | | 7 | (43.8%, 95% CI 19.4~68.1; 7/16) | |
| Likelihood of authenticity of government supported projects, TCM vs CM | | | | | RR 1.24, 95%CI 0.67~2.30; P=0.49; (25/46)/(7/16) | | | | | |
| Level 3 hospitals or medical institutes | | | | | | | | | | |
| Self-described RCT | | 495 | | (15.8%, 495/3137) | 192 | (13.2%, 192/1452) | | 303 | (18.0%, 303/1685) | |
| Multiple versions of publication | | 27 | | (5.5%, 27/495) | 7 | (3.6%, 7/192) | | 20 | (6.6%, 20/303) | |
| Author could not be contacted | | 103 | | (22.0%, 103/(495−27)) | 28 | (15.1%, 28/(192−7)) | | 75 | (26.5%, 75/(303−20)) | |
| Author refused to answer | | 13 | | (2.8%, 13/(495−27)) | 6 | (3.2%, 6/(192−7)) | | 7 | (2.5%, 7/(303−20)) | |
| Authentic RCTs | | 55 | | (11.8%, 95% CI 8.8~14.7; 55/(495−27)) | 23 | (12.4%, 95% CI 7.7~17.2; 23/(192−7)) | | 32 | (11.3%, 95% CI 7.6~15.0; 32/(303−20)) | |
| Likelihood of authenticity, TCM vs CM | | | | | RR 1.10, 95% CI 0.67~1.82; P=0.71; (23/(192−7))/(32/303−20)) | | | | | |
| Study concerned pre-market drug | | 10 | |  | 5 | | | 5 | | |
| Study concerned pre-market drug and was authentic RCT | | 10 | | (100%, 10/10) | 5 | (100%, 5/5) | | 5 | (100%, 5/5) | |
| Study was funded by government or other official source | | 16 | |  | 6 |  | | 10 |  | |
| Study was funded by government or other official source and was authentic RCT | | 9 | | (56.3%, 95% CI 32~81.0; 9/16) | 6 | (100%, 6/6) | | 3 | (30.0%, 95%CI1.6~58.4; 3/10) | |
| Level 2 hospitals and below | | | | | | | | | | |
| Self-described RCT | 1929 | | | (61.5%, 1929/3137) | 929 | (64.0%, 929/1452) | | 1000 (59.3%, 1000/1685) | | |
| Multiple versions of publication | 26 | | | (1.3%, 26/1929) | 14 | (1.5%, 14/929) | | 12 | (1.2%, 12/1000) | |
| Author could not be contacted | 470 | | | (24.7%, 470/(1929−26)) | 209 | (22.8%, 209/(929−14)) | | 261 | (26.4%, 261/(1000−12)) | |
| Author refused to answer | 53 | | | (2.8%, 53/(1929−26)) | 17 | (1.9%, 17/(929−14)) | | 36 | (3.6%, 36/(1000−12)) | |
| Authentic RCTs | 24 | | | (1.3%, 95% CI 0.8~1.8; 24/(1929−26)) | 11 | (1.2%, 95% CI 0.5~1.9; 11/(929−14)) | | 13 | (1.3%, 95% CI 0.6~2.0; 13/(1000−12)) | |
| Likelihood of authenticity, TCM vs CM | | | | | RR 0.91, 95% CI 0.41~2.03; P=0.82; (11/(929−26))/(13/(1000−12)) | | | | | |
| Study concerned pre-market drug | | | 1 | | 1 |  | | 0 |  | |
| Study concerned pre-market drug and was authentic RCT | | | 1 | | 1 |  | | 0 |  | |
| Study was funded by government or other official source | | | 1 | | 1 |  | | 0 |  | |
| Study was funded by government or other official source and was authentic RCT | | | 1 | | 1 |  | | 0 |  | |
| All institutions | | | | | | | | | | |
| Self-described RCT | 3137 | | |  | 1452 | |  | 1685 | |  |
| Multiple versions of publication | 83 | | | (2.6%, 83/3137) | 32 | | (2.2%, 32/1452) | 51 | | (3.0%, 51/1685) |
| Author could not be contacted | 735 | | | (24.1%, 735/(3137−83)) | 312 | | (22.0%, 312/(1452−32)) | 423 | | (25.9%, 423/(1685−51)) |
| Author refused to answer | 84 | | | (2.8%, 84/(3137−83)) | 35 | | (2.5%, 35/(1452−32)) | 49 | | (3.0%, 49/(1685−51)) |
| Authentic RCTs | 207 | | | (6.8%, 95% CI 5.9~7.7; 207/(3137−83)) | 103 | | (7.3%, 95% CI 5.9–8.7; 103/(1452−32)) | 104 | | (6.4%, 95% CI 5.2~7.6; 104/(1685−51)) |
| Likelihood of authenticity, TCM vs CM |  | | |  | RR 1.14, 95% CI 0.88~1.46; P=0.33; (103/(1452−32))/(104/(1685−51)) | | | | | |
| Author understood randomisation principles but claimed non-RCT as RCT | 115 | | | (5.1%, 95%CI 4.2~6.0; 115/(3137−83−735−84)) | 88 | | (8.2%, 95%CI 6.6~9.8; 88/(1452−32−312−35)) | 27 | | (2.3%, 95%CI 1.4~3.2; 27/(1685−51−423−49)) |
| Author did not know randomisation principles well and incorrectly claimed non-RCT as RCT | 1913 | | | (85.6%, 95%CI 84.1~87.1; 1913/(3137−83−735−84)) | 882 | | (82.2%, 95%CI 79.9~84.5; 882/(1452−32−312−35) | 1031 | | (88.7%, 95% 86.9~90.6; 1031/(1685−51−423−49)) |
| Comparative rates of authenticity by type of institution | | | | | | | | | | |
| Medical university and college affiliated hospitals vs level 3 hospitals and medical institutes | | | RR 1.58, 95% CI 1.18~2.13; (128/683)/(55/468), P=0.002 | | RR 1.73, 95% CI 1.12~2.68, (69/320)/(23/185); P=0.01) | | | RR 1.44, 95% CI 0.96~2.15, (59/363)/(32/283); P=0.08 | | |
| Medical university and college affiliated hospitals vs level 2 and lower hospitals | | | RR 14.42, 95% CI 9.40~22.10; (128/683)/(24/1903), P<0.00001 | | RR 17.94, 95%CI 9.62~33.46, (69/320)/(11/915); P<0.00001 | | | RR 12.35, 95% CI 6.86~22.25, ((59/363)/(13/988), P<0.00001 | | |
| Level 3 hospitals and medical institutes vs level 2 and lower hospitals | | | RR 9.32, 95% CI 5.83~14.89; (55/468)/(24/1903), P<0.00001 | | RR 10.34, 95%CI 5.13~20.84, (23/185)/(11/915), P<0.00001 | | | RR 8.59, 95% CI 4.57~16.15, (32/283)/(13/988) | | |

Footnotes: TCM=Traditional Chinese Medicine, CM=conventional medicine
